# GLUTATHIONE, INFLAMMATION, MITOCHONDRIAL FAT OXIDATION AND DIASTOLIC HEART FUNCTION IN OLD MICE

**DOI:** 10.1093/geroni/igz038.1553

**Published:** 2019-11-08

**Authors:** George E Taffet, K A Cieslik, R V Sekhar, Celia A Pena Heredia, Dale J Hamilton, Mark L Entman

**Affiliations:** 1 Baylor College of Medicine, Houston, Texas, United States; 2 Houston Methodist Hospital, Houston, Texas, United States

## Abstract

Impaired diastolic function is a risk factor for diastolic heart failure, may limit exercise performance, and is common in aging in both people and animals. This diastolic dysfunction seems to be associated with cardiac inflammation, fibrosis and impaired mitochondrial fatty acid metabolism. Old (24-28 m) mice fed a GlyNAC supplemented diet for 8 weeks were compared to those on control diet, and had dramatic improvement in all these parameters. For example, ATP generation from fatty acids with five-fold higher in the GlyNAC supplemented mice. In vitro studies compared NAC with GlyNAC and demonstrated the benefits only with supplementing both amino acids as compared to NAC alone. These data suggest that GlyNAC may have a role in improving cardiac function thus improving exercise tolerance and quality of life for older people.

